# ESBL- and pAmpC-producing Enterobacterales from Swedish dogs and cats 2017–2021: a retrospective study

**DOI:** 10.1186/s13028-024-00786-2

**Published:** 2025-01-06

**Authors:** Anna Bonnevie, Mattias Myrenås, Oskar Nilsson

**Affiliations:** 1 Department of Animal Health and Antibiotic Strategies, Swedish Veterinary Agency, Uppsala, Sweden; 2 Department of Clinical Sciences, Swedish University of Agricultural Sciences, Uppsala, Sweden

**Keywords:** Antibiotic resistance, Antimicrobial resistance, Companion animals, Extended-spectrum beta-lactamases, Plasmid-mediated AmpC

## Abstract

**Background:**

Antibiotic resistant bacteria are a threat to both human and animal health. Of special concern are resistance mechanisms that are transmissible between bacteria, such as extended-spectrum beta-lactamases (ESBL) and plasmid-mediated AmpC (pAmpC). ESBL/AmpC resistance is also of importance as it confers resistance to beta-lactam antibiotics including third generation cephalosporins. The Swedish Veterinary Agency (former English name National Veterinary Institute) performs confirmatory testing of suspected ESBL-/pAmpC-producing Enterobacterales. The aim of this study is to describe the clinical background, antibiotic susceptibility, and genetic relationships of confirmed isolates from dogs and cats in Sweden from 2017 to 2021.

**Results:**

The study includes 92 isolates of ESBL/pAmpC-producing bacteria from 82 dogs, and 28 isolates from 23 cats. *Escherichia coli* was the most commonly isolated bacteria, and the most frequent sampling site was the urinary tract. From eight dogs and two cats, ESBL/pAmpC-producing bacteria were isolated on more than one occasion. Multi-resistance was more than twice as common in samples from dogs (50%) than in samples from cats (22%). Among dogs, sequence type (ST) 131 and ST372 were the dominant strains and *bla*_CMY-2_ and *bla*_CTX-M-15_ the dominant genes conferring reduced susceptibility to third-generation cephalosporins. Among cats, ST73 was the dominant strain and *bla*_CTX-M-15_ the dominant gene.

**Conclusions:**

Monitoring the resistance patterns and genetic relationships of bacteria over time is important to follow the results of measures taken to reduce resistance. Knowledge of the appropriate antibiotic usage is also crucial. In this study, a variety of STs and ESBL/pAmpC-genes were detected among the isolates. There were available antibiotics likely effective for treatment in all cases, based on resistance pattern, infection site and host species.

## Background

Bacteria that are resistant to antibiotics are a large threat to public health worldwide and they are estimated to be associated with almost five million human deaths per year [1].

Antibiotic resistant bacteria are not only a human health problem. Bacteria that can be transmitted between humans, animals and the surrounding environment constitute an important one-health concern [2, 3]. Infections by resistant bacteria in animals might also have a direct negative impact on animal health and welfare, due to therapeutic failure and prolonged suffering [4]. There may also be negative economic and emotional consequences for the owner of the animal [4].

The inappropriate use of antibiotics in humans as well as in animals and the transmission of resistant pathogens among humans, animals and environment are the main drivers of antibiotic resistance development [5]. The burden of antibiotic resistant bacteria differs among countries. Multi-disciplinary strategies are required to decrease antibiotic resistance, including legislation, monitoring of resistance, education of prescribers and public, Antimicrobial Stewardship Programs et cetera [6]. Regulations at the EU (Regulation (EU) 2019/6) and at the national level (SJVFS 2019:32) as well as the EU Farm to Fork strategy within the European Union Green Deal aim to reduce antibiotic usage. As further support for prescribing veterinarians, there are several guidelines and recommendations to favour prudent antibiotic usage [7–10]. By combining guidelines with local knowledge about resistance patterns and prevalent genotypes in the current area, veterinarians can further improve their antibiotic use, which is essential for preventing the selection of resistant bacteria.

There are different mechanisms of resistance, some of which are transferable between bacteria. One of the most important variants in human medicine is resistance to extended spectrum cephalosporins among Enterobacterales, such as *Escherichia coli* and *Klebsiella* spp. [1]. When this resistance is transferable due to the production of extended-spectrum beta-lactamases (ESBL) and/or plasmid-mediated AmpC (pAmpC), it is considered particularly problematic. In these cases, not only might the bacteria spread to other animals or humans, but the resistance mechanism can also be spread to other bacteria, through gene transfer. In Sweden, ESBL-/pAmpC-producing Enterobacterales are notifiable when detected in humans (SFS 2004:168), but not when detected in samples from animals. However, special emphasis has been placed on this type of resistance within the Swedish veterinary antibiotic resistance monitoring programme (Svarm). The Swedish Veterinary Agency (SVA) performs confirmatory testing of suspected ESBL-/AmpC-producing Enterobacterales sampled from animals, funded by The Swedish Board of Agriculture. The first confirmed cases in dogs and cats were reported in 2008 and 2009, respectively [11].

The aim of this study is to describe in detail the isolates of ESBL-/pAmpC-producing Enterobacterales from dogs and cats, confirmed at SVA during 2017–2021. The types of infections associated with the isolates, the antibiotic susceptibility, and the genetic relationships among the isolates are described.

## Methods

### Isolates

Diagnostic veterinary laboratories in Sweden are encouraged to submit clinical isolates of Enterobacterales with suspected resistance to extended spectrum cephalosporins to SVA for confirmatory testing and subtyping. These investigations are funded by the Swedish Board of Agriculture and are free of charge for the referring laboratories. In this study, all the submitted isolates confirmed as ESBL-/pAmpC-producing Enterobacterales between 1 January 2017 and 31 December 2021 were included.

The isolates were described regarding the origin of sampling, bacterial species, pattern of antibiotic resistance and genetic profile for dogs and cats, respectively. Repeated isolates from the same animal were compared to the first isolate and the time interval between sampling was noted.

For isolate species determined by matrix-assisted laser desorption/ionisation time-of-flight mass spectrometry (MALDI-TOF MS, Bruker Daltonics, Bremen, Germany) as belonging to the *Enterobacter cloacae* group (n = 5), species determination was further refined by using KmerFinder version 3.2 with database version 2022-07-11 on de novo genome assemblies [12–14].

### Sampled animals and reason for sampling

The sampled animals were described regarding species, breed, and sex. Each isolate was categorized in one of the following sampling site groups: the urogenital system, wounds, and other sites. The clinical signs related to the isolates were described based on the information provided on the referral note.

### Antimicrobial susceptibility testing

Antimicrobial susceptibility testing was performed using broth micro-dilution method on custom made panels (Thermo Fisher Scientific, Waltham, MA USA), testing antibiotics relevant in the clinical veterinary setting within Sweden. Reference strains *Escherichia coli* ATCC 25922 and *Acinetobacter baumannii* 2012-70-100-69 (used for control of higher concentrations of cephalosporins and carbapenems) were included and evaluated as quality controls of performed susceptibility tests at least once weekly. All the isolates were assessed for susceptibility to ampicillin, amoxicillin-clavulanic acid, cefotaxime, colistin, enrofloxacin, gentamicin, neomycin, nitrofurantoin, tetracycline, trimethoprim-sulphamethoxazole and meropenem. The results of the susceptibility testing were interpreted according to epidemiological cut-off values (ECOFFs). ECOFFs classify isolates with acquired reduced susceptibility as non-wild type, which is referred to as resistant in this paper. Resistance against beta-lactams is part of the definition of ESBL-/AmpC-production. However, assessment of susceptibility to other substances is hampered because ECOFFs are often not defined for bacterial species other than *E. coli*. When no ECOFF was available for the specific combination of bacteria and substance, resistance was assessed by comparison with distribution tables available from European Committee on Antimicrobial Susceptibility Testing (EUCAST) or, alternatively, with the ECOFF for *E. coli* (Table 1). Cut-off values deviating from the ones for *E. coli* were for *Enterobacter* spp. cefotaxime (1 mg/L) and tetracycline (16 mg/L) and for *Proteus mirabilis* gentamicin (4 mg/L) and tetracycline (16 mg/L).

**Table 1:**
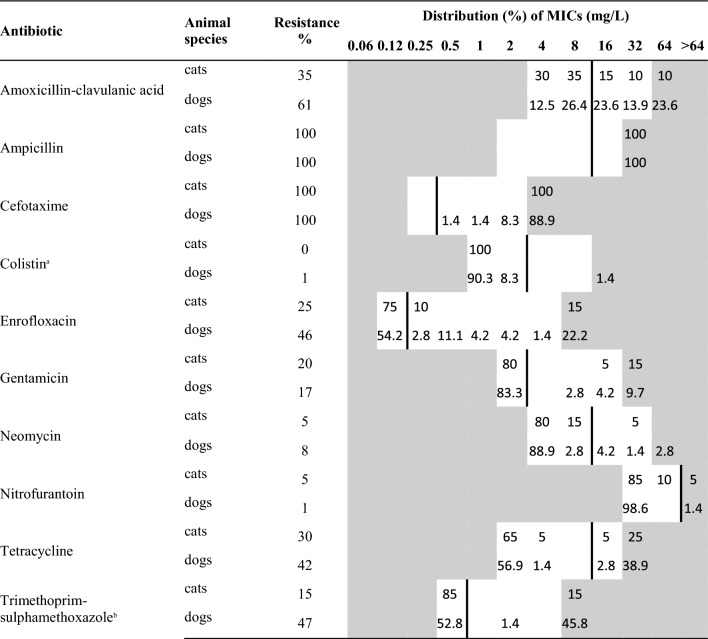
Distribution of MICs and resistance in ESBL/pAmpC-producing *Escherichia coli* from cats (n = 20) and dogs (n = 72)

White fields denote test ranges for each antibiotic substance. The percentage of isolates with a certain MIC of an antibiotic substance is given in the corresponding field. Percentages shown above the highest test concentration represent isolates with a MIC > the highest test concentration. Numbers shown in the lowest test concentration represent isolates with a MIC ≤ the lowest test concentration. Vertical bold lines indicate cut-off values used to define resistance

^a^The isolate with MIC 16 mg/L was investigated for mcr-genes and found negative

^b^Concentration of trimethoprim given, tested in concentration ratio 1/20 (trimethoprim-sulphamethoxazole)

### Confirmatory testing

All the included isolates were species identified using MALDI-TOF MS. Subsequently, phenotypic confirmatory tests for the production of ESBL or AmpC in isolates confirmed as Enterobacterales were performed in Sensititre EUVSEC2 microdilution panels (Thermo Fisher Scientific, Waltham, MA, USA) and interpreted according to EUCAST.

Isolates with an AmpC phenotype were subjected to polymerase chain reaction (PCR) to detect transferable genes encoding AmpC [15] and ESBL [16, 17]. Isolates with a transferable gene encoding AmpC and isolates with an ESBL phenotype were subjected to genome sequencing.

### Genome analysis

DNA was extracted from overnight cultures on horse-blood agar using an EZ1 DNA tissue kit (Qiagen, Halden, Germany). For a subset of ESBL-producing Enterobacterales, DNA was extracted by using an IndiMag Pathogen Kit (Indical Bioscience) in a Maelstrom 9600 (TANBead). DNA concentrations were determined using a Qubit HS DNA-kit (Life Technologies) and subsequently sent to Clinical Genomics Stockholm, SciLifeLab (Solna, Sweden) for library preparation and paired-end sequencing via Illumina technology. Reads were trimmed using Trimmomatic v0.34, and the specific ESBL-gene was determined using “Antimicrobial Resistance Identification By Assembly (ARIBA)” [18] against the Resfinder (https://cge.cbs.dtu.dk/services/ResFinder/) database. Genome assembly was performed with SPAdes v3.14.0 with the “careful” parameter, followed by Pilon v1.23 [19] with default settings to correct the assemblies. Using the assembled contigs, the isolates were assigned a multi-locus sequence type (MLST) when available, using Ridom SeqSphere + v9.0 software (Ridom GmbH, Germany). *E. coli* MLST was extracted from the material using the Achtman scheme [20], also in SeqSphere + .

The assembled contigs were analysed using the 2,513 loci standardized Escherichia/Shigella v1 core-genome MLST (cgMLST) scheme from Enterobase [21] in SeqSphere + with the parameters 70% identity and 100% coverage. Using the results of the cgMLST, phylogenetic distance was calculated using the Neighbour-joining tree method [22] in SeqSphere + , pairwise ignoring missing values, and the resulting trees were visualized in iTol v6 [23].

## Results

In total, 92 isolates of ESBL/pAmpC-producing bacteria from 82 dogs, and 28 isolates from 23 cats were confirmed. Of these 120 isolates, the most commonly cultured bacterial species was *E. coli* (n = 104). The remaining isolates were *Klebsiella pneumoniae* (n = 8), *Enterobacter hormaechei* (n = 3), *Proteus mirabilis* (n = 2), *Enterobacter kobei* (n = 1), *Enterobacter roggenkampii* (n = 1), and *Klebsiella oxytoca* (n = 1). For 68 of the isolates, information about clinical disease as a reason for sampling the individual animals was available in the referral notes.

### Isolates from dogs

**Fig. 1:**
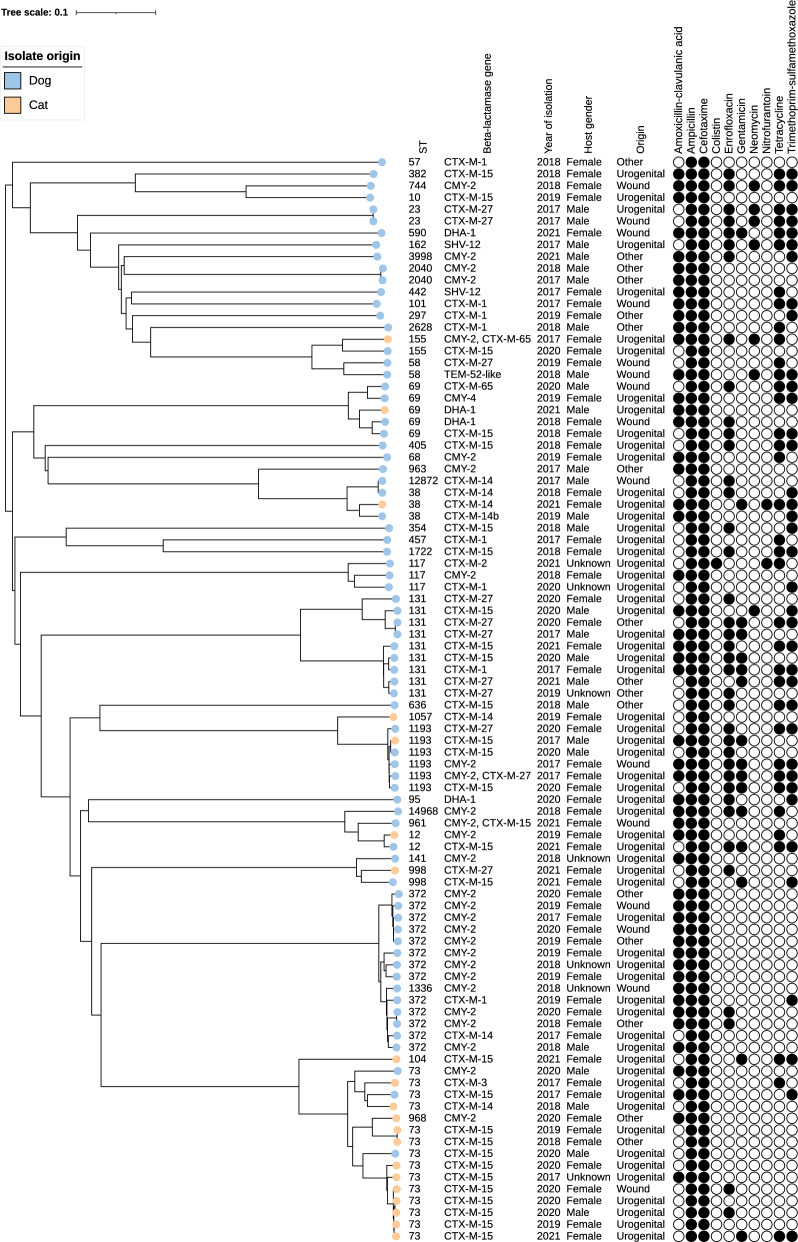
Dendrogram from cgMLST data of ESBL-/pAmpC- producing *Escherichia coli* from dogs and cats. Dataset consisting of 2513 loci showing 92 isolates of ESBL-/pAmpC- producing *Escherichia coli* from 20 cats and 72 dogs confirmed at the Swedish Veterinary Agency 2017–2021. Sequence type (ST, Achtman), β-lactamase gene, year of isolation, host species, host sex, sample origin, and phenotypic resistance towards antibiotic substances indicated as minimum inhibitory concentration (MIC) above (full circles) or equal to/below (empty circles) EUCAST epidemiological breakpoints. Repeated samples from the same individual were not included

Isolates of ESBL/pAmpC-producing bacteria from 82 dogs were included in the study. The majority of the isolates were *E. coli* (n = 72) (Fig. 1). The remaining isolates consisted of *K. pneumoniae* (n = 4), *E. hormaechei* (n = 3), *P. mirabilis* (n = 2), and *K. oxytoca* (n = 1). The two most common ESBL/pAmpC genes among the isolates were *bla*_CMY-2_ and *bla*_CTX-M-15_, which were detected in more than half of the isolates. Among the remaining isolates, *bla*_CTX-M-27_ and *bla*_CTX-M-1_ were the most common genes.

Forty different breeds were represented, and except for mixed breed (n = 9), no breed was represented more than three times. There were more bitches (n = 49) than males (n = 25). Information of sex was not available for eight dogs. The ages ranged from two months to 17 years, with a median of seven years. Most of the dogs were between one and ten years old (n = 44), fewer were older than 10 years (n = 12) or younger than one year old (n = 7). For 19 dogs, information about age was not available.

The most common sampling site was the urogenital tract (n = 50). Forty-eight of these were urinary samples and two were vaginal samples. Forty-two of the 48 urinary isolates and both vaginal isolates were cultured as *E. coli*. The remaining urinary isolates were *K. pneumoniae* (n = 2), *P. mirabilis* (n = 2), *K. oxytoca* (n = 1), and *E. hormaechei* (n = 1).

The main clinical reason for retrieving urogenital samples was signs of urinary tract infection (UTI) (n = 15). Other reasons included stranguria in one dog, glucosuria in one lethargic dog treated for diabetic mellitus, haematuria in one paralysed dog, clinical signs of polyuria/polydipsia in one dog and suspected pyelonephritis in one dog. In one dog, the clinical reason for sampling the urinary tract was not available, but the dog had previously been diagnosed with ESBL-/pAmpC-producing bacteria. In one dog, recurrent purulent vaginitis was the cause of sampling.

The second largest group of isolates originated from wounds (n = 16), including twelve samples from postoperative wounds. The types of surgeries preceding infection were ovariohysterectomy due to pyometra (n = 3), enterotomy (n = 2), anal sac extirpation (n = 1), reconstruction of perineal hernia (n = 1), tibial plateau levelling osteotomy (n = 1) and non-specified surgery (n = 4). The non-surgical wounds included bite wound (n = 1), ruptured mass (n = 1) and wounds of unknown origin (n = 2). From wound isolates, ESBL/pAmpC-producing *E. coli* (n = 14), *K. pneumoniae* (n = 1), and *E. hormaechei* (n = 1) were cultured.

The remaining isolates (n = 16) originated from other anatomical sampling sites. Isolates originating from the mammary gland (n = 4) were sampled due to suspected mastitis or obstructed milk ducts in three dogs. There was no information about clinical indication for the last dog. Isolates originating from respiratory tract (n = 3) included one dog with pneumonia and one dog with thoracic cavity effusion, and the clinical signs were not noted for one dog. Isolates originating from abdominal cavity (n = 3) included one abscess, one caecal mass and one free fluid aspirate. Isolates from ear (n = 3) were sampled due to unilateral pain in the ear for one dog, while reason was not noted for two dogs. From each of the following sampling sites, one isolate each was retrieved: from the cornea due to corneal ulceration, from the anal sac due to anal sacculitis and from faeces due to diarrhoea. In isolates from other sampling sites than urogenital tract and wounds, ESBL/pAmpC-producing *E. coli* (n = 14), *K. pneumoniae* (n = 1, mammary gland) and *E. hormaechei* (n = 1, respiratory tract) were cultured.

The sampling site, genomic variation, and antimicrobial susceptibility of the isolates cultured as *E. coli* are listed in Fig. 1.

Using available information regarding ECOFFs, the most common finding among all the isolates from dogs were resistance against trimethoprim-sulphonamides (52%), enrofloxacin (50%) and tetracycline (43%). Resistance to gentamicin was also a common trait (24%), whereas resistance to neomycin (9%), nitrofurantoin (7%) and colistin (2%) was more uncommon. Resistance to meropenem was not detected in any of the isolates. Half of the isolates (50%) were resistant to at least two substances in addition to beta-lactams, i.e., multi-resistant. In contrast, approximately one-fourth of the isolates (27%) were resistant to beta-lactams only. The occurrence of resistance and proportion of multi-resistant strains when assessing only isolates of *E. coli* (Table 1) were comparable to the levels among all isolates in the study. Among the isolates of *E. coli* with an ESBL-gene, 15 of 43 (35%) were resistant to amoxicillin in combination with clavulanic acid.

The 72 *E. coli* isolates were separated into 37 different sequence types (STs) (Fig. 1). The most common strains were ST372 (n = 13) and ST131 (n = 9). Furthermore, one isolate with ST1336 clustered together with the other ST372 isolates. A total of 14 different ESBL/pAmpC-genes or gene combinations were present among the 72 *E. coli* isolates. The most common were *bla*_CMY-2_ (n = 23) and *bla*_CTX-M-15_ (n = 17).

The most common combination of STs and genes was ST372 and the gene *bla*_CMY-2_ (n = 11). In addition, the ST1336 isolate also carried this gene. The remaining 60 isolates were separated into 51 different combinations of STs and ESBL/pAmpC-genes. The most common ST131 gene polymorphism was *bla*_CTX-M-27_, which occurred in five isolates.

### Isolates from cats

Isolates with ESBL/pAmpC-producing bacteria from 23 cats were included in the study.

As for dogs, the majority of the isolates were *E. coli* (n = 20), the remaining isolates were *E. kobei* (n = 1), *E. roggenkampii* (n = 1) and *K. pneumoniae* (n = 1). The most common ESBL/pAmpC gene was *bla*_CTX-M-15_ (Fig. 1). This gene was detected in almost half of the isolates.

A total of six different cat breeds were included, and except for mixed breed (n = 8), Oriental Shorthair was the most common breed (n = 4), followed by Sphynx (n = 3) and Birman (n = 2). Fifteen cats were females, seven were males and for one cat, sex was not noted. The age ranged between three months and 19 years, with a median of three years. Two cats were younger than one year old, eleven were between one and ten years old and eight were older than ten years old. For two cats, age was not noted.

The most common sampling site was the urogenital tract (n = 20), of which 16 were urine samples and four vaginal samples. Isolates from urine were *E. coli* (n = 13), *E. kobei* (n = 1), *E. roggenkampii* (n = 1) and *K. pneumoniae* (n = 1). All the isolates from the vagina were *E. coli*. The clinical indications for retrieving urinary samples were urinary tract inflammation or infection (n = 7), haematuria (n = 1), and diarrhoea (n = 1). For seven cats, information about sampling indication was not available. The retrieval of samples from the vagina was due to yellow vaginal discharge in one cat, for the other three cats information was lacking.

The isolates from other origins than the urogenital tract were obtained from mammary gland (n = 1), mammary gland tumour (n = 1) and postoperative infected wound (n = 1). All three of these isolates were *E. coli.*

The sampling site, genomic variation, and antimicrobial susceptibility of the isolates cultured as *E. coli* are listed in Fig. 1.

The most common resistances among the isolates from cats were to enrofloxacin (26%) and tetracycline (26%). Resistance to gentamicin (17%) and trimethoprim-sulphonamides (13%) were also common traits, whereas resistance to colistin (4%), neomycin (4%), and nitrofurantoin (4%) was detected only in one isolate, respectively. Resistance to meropenem was not detected in any of the isolates. Approximately one-fifth of the isolates (22%) were resistant to at least two substances apart from beta-lactams, i.e. multi-resistant. In contrast, approximately half of the isolates (48%) were resistant to beta-lactams only. The occurrence of resistance and proportion of multi-resistant strains when assessing only isolates of *E. coli* (Table 1) were comparable to the levels among all isolates in the study. Among the isolates of *E. coli* with an ESBL-gene, 3 of 16 (19%) were resistant to amoxicillin in combination with clavulanic acid.

The 20 *E. coli* isolates from cats were separated into 10 different STs (Fig. 1). The most common was ST73, identified in 11 of the 20 isolates. Furthermore, one isolate with ST968 clustered together with these other isolates. A total of seven different ESBL/pAmpC-genes or gene combinations were present among the 20 *E. coli* isolates. The most common gene was *bla*_CTX-M-15_ (n = 11).

The most common combination of STs and genes was ST73 and the gene *bla*_CTX-M-15_ (n = 9). The remaining eleven isolates all had separate combinations of STs and ESBL/pAmpC-genes.

On two occasions, cats in the same households were found to carry closely related isolates. From one household, two Oriental shorthairs sampled from the vagina in 2019 and 2021 respectively carried closely related *E. coli* (ST73 and *bla*_CTX-M-15_). In another household, two Sphynx cats were sampled from the vagina and from a mammary gland tumour in 2018 and 2019 respectively, both carrying closely related *E. coli* (ST73 and *bla*_CTX-M-15_).

### Individuals with multiple isolates

From eight dogs and two cats, ESBL/pAmpC-producing bacteria were isolated on more than one occasion. The dogs were sampled on two (n = 7) and four (n = 1) separate occasions respectively, whereas the two cats were sampled on three (n = 1) and four (n = 1) occasions. The time from the first to the last recorded sampling date was between one and 15 months in dogs and between eight and nine months in cats. The sampling site was the same for all isolates from the same animal, except for one dog, where the first isolate was from the urinary tract and the second sample from the anal gland. The bacteria isolated from all the dogs and one of the cats were *E. coli*, whereas from the second cat the bacteria was *K. pneumoniae*.

In all the cases where ESBL/pAmpC-producing bacteria were isolated on more than one occasion, the same combination of bacterial species and ESBL/pAmpC gene was detected. However, in the dog where the isolates came from two different anatomical sites of the animal, the isolates were of different STs.

Furthermore, the resistance profiles of the isolates from each individual animal varied on four occasions (two cats and two dogs). Resistance to a specific substance (enrofloxacin, gentamicin, neomycin, tetracycline, trimethoprim-sulphonamides) disappeared on nine occasions whereas resistance to a specific substance (gentamicin, trimethoprim-sulphonamides) appeared on three occasions. From one cat there were three isolates included in the study, where the isolate from the second sampling occasion had a different resistance profile than the isolates from the first and the third sampling.

## Discussion

Between 2017 and 2021, ESBL/pAmpC-producing bacteria were confirmed at SVA in 92 isolates from dogs and 28 isolates from cats. As the suspected isolates were sent to SVA for confirmation from many different laboratories, it is unknown how many isolates, not suspected to be ESBL/pAmpC-producing, that were investigated at the different laboratories. Thereby, it is not possible to assess the proportion of isolates that were ESBL/pAmpC-producing. However, about half of the confirmed isolates were from the diagnostic laboratory at SVA. For this subset, the proportion of ESBL/pAmpC-producing bacteria among all investigated Enterobacterales was approximately 0.5% for both dogs and cats respectively (data not shown). The most common sampling site for both dogs and cats was the urogenital tract, and *E. coli* was the most commonly cultured bacteria. These results are consistent with expectations based on previous reports [24–26]. Bacterial UTIs are considered common in dogs and less common in cats [10]. Regardless of the bacterial species, the choice of antibiotic for treatment of lower UTIs might differ from that for other anatomical sites due to drug accumulation in the urinary bladder and, subsequently, high antibiotic concentrations in the urine. This is especially true for antibiotics that are excreted via urine, such as beta-lactams, trimethoprim-sulphonamides, nitrofurantoin, and gentamicin. The frequency of genes associated with reduced susceptibility to third-generation cephalosporins was comparable to what have been reported in studies from other European countries [27–34], with some variations. A study on imported dogs to Finland [27] during 2017–2018 found proportionally less *bla*_CMY-2_ and more *bla*_CTX-M-15_ than our study, while a study on UTI from cats and dogs in Portugal [28] 1999–2015 found more *bla*_CMY-2_ and less *bla*_CTX-M-15_. We did not find *bla*_CTX-M-55_ in our sample set, a gene which was the third most common ESBL gene found in a study on ESBL in urine samples from Swiss cats and dogs 2012–2016 [29] and which was also the third most common ESBL gene in a study on ESBL and pAmpC in companion animals in the UK 2010–2016 [34].

In addition to legislative regulations to restrict the use of antibiotics, there are national and international guidelines for supporting veterinarians in their treatment decisions [7–10]. The guidelines highlight the importance of bacterial culture and susceptibility testing in suspected bacterial UTIs. However, in dogs with sporadic cystitis, empirical therapy without preceding bacterial culture and susceptibility testing is considered standard practice. Amoxicillin without clavulanic acid is recommended as an empirical treatment for lower UTIs according to both Swedish and international recommendations [7, 9, 10]. Trimethoprim-sulphonamides are the secondary choice and nitrofurantoin is the third option according to international guidelines [10]. Treatment with trimethoprim-sulphonamide might be associated with greater adverse effects than amoxicillin [9, 10]. Nitrofurantoin has been suggested to be reserved for cases where amoxicillin, with or without clavulanic acid, and trimethoprim-sulphonamide are not suitable based on susceptibility testing or due to comorbidities in the patient [10]. There are limited published data about the use of nitrofurantoin for treating UTIs in dogs and cats [35, 36]. One study reported successful outcome in twelve of 14 dogs treated with nitrofurantoin for UTIs, but also treatment failure due to progressive nitrofurantoin resistance in two dogs [36].

In this study, isolates with MIC above ECOFFs, i.e. isolates with acquired reduced susceptibility, are referred to as resistant. It should be noted that defining resistance by the use of ECOFFs might not always be consistent with clinical resistance. In order to properly assess clinical resistance, clinical breakpoints are needed. However, such breakpoints are often missing in veterinary medicine. ESBL/AmpC-production of the investigated isolates excludes effective treatment with amoxicillin without clavulanic acid, which is the first line treatment for lower UTIs, however, amoxicillin in combination with clavulanic acid could have been effective treating 30% of the cases of UTI in dogs in this study. Trimethoprim-sulphonamides could have been effective in approximately 45% of the dog UTIs and nitrofurantoin could have been effective in all dog UTIs. Gentamicin could have been effective in approximately 75% of UTIs in dogs. Since it is eliminated through glomerular filtration, it seems to be an appropriate drug; however, the potentially nephrotoxicity profile of this drug makes it a suboptimal choice in most clinical cases [37, 38]. Enrofloxacin could have been effective in 47% of dog UTIs but was not the only effective substance in any of the cases. Enrofloxacin is not a suitable choice for treatment of ESBL/pAmpC-producing bacteria in lower UTIs for several reasons. The substance is eliminated mainly via faeces and only a small portion through urine, making it less efficient for treatment of lower UTIs [39]. Apart from this, since 2013, national regulations states that this substance can only be prescribed to animals when no other antibiotic treatment is suitable according to resistance pattern or in severely ill animals, where delay of treatment awaiting susceptibility results is dissuaded (SJVFS 2019:32). Therefore, there would be no indication to use enrofloxacin in the described cases. The fact that more than half of the isolates showed resistance to enrofloxacin is notable as fluoroquinolones are seldomly used in veterinary medicine in Sweden [11]. The occurrence of resistance is also much higher than among all clinical isolates of *E. coli* from urinary samples of dogs (3–6%) and cats (4–7%) reported in 2017–2021 in the Swedish monitoring program Svarm [11]. The reason for this is not known but co-existence of additional resistance genes apart from the ESBL/pAmpC genes on the same plasmids is one potential explanation that merits further investigations.

The number of isolates from sites other than the urogenital tract was too small to draw conclusions about possible treatment outcomes based on susceptibility to different substances.

The overall multi-resistance, defined as resistance to at least two substances other than beta-lactams, was 44%. The percentage of multi-resistant bacteria among the dog isolates (50%) was more than twice the percentage among the cat isolates (22%). Interspecies dissimilarity was also detected in terms of resistance to individual substances. The variation may reflect differences in the epidemiology of the isolates, i.e., the original source from which the animals were infected. Differences in antibiotic usage may also exist between the two species due to the characteristic spectra of diseases. In dogs, bacterial UTIs are common, whereas aseptic inflammation is more common in cats with clinical signs of disease in the lower urinary tract [10]. Likewise, atopic skin disease with secondary bacterial pyodermatitis is common in dogs, whereas this condition presents differently in cats and therefore might go unrecognized [40, 41]. The medical reasons for prescribing antibiotics thus differs between the species. To the best of our knowledge, there are no information available regarding owner compliance and the actual administred dose of antibiotics for dogs and cats in Sweden.The difference in resistance among ESBL/pAmpC producing bacteria between the two animal species is probably not due to selection by the use of antibiotics in dogs and cats per se. Instead, more frequent use of antibiotics could increase the probability of ESBL/pAmpC producing bacteria transiently colonizing the host, when non-resistant bacteria are decreased during treatment. Furthermore, the use of substances other than beta-lactams could increase the likelihood that multi-resistant variants of ESBL/pAmpC producing bacteria colonize the animals.

Bacterial infection is not always an indication for antibiotic treatment, which is emphasized in guidelines and consensus documents [7, 8]. For example, surgical debridement and cleaning of wounds, together with other supportive treatments, can be enough to clear infection. By treating comorbidities and conditions predisposing individuals to infection, the incidence of bacterial infections can be reduced. The overall incidence of bacterial infections can be minimized by maintaining good surgical practices and maintaining high hygienic standards in veterinary practice facilities.

In this study, the most common *E. coli* ST was ST73, which was isolated mainly from cats. Furthermore, one cat isolate with ST968 differed from ST73 in only one locus and belonged to the same clonal cluster (CC73). ST73 is often found among the most common STs in human clinical non-ESBL isolates. It has also been found to be prominent in cats, in studies including non-ESBL isolates [42, 43]. Isolates belonging to ST73 have also been found in Australian and French canine pathogenic isolates [44, 45]. In this study, there were three ST73 isolates originating from dogs. The second most common ST was ST372, which was found only in isolates from dogs. One isolate from a dog with ST1336 clustered with the ST372 isolates, differing in only one locus. ST372 has previously been shown to be associated with dogs but is more seldom found in humans, with local exceptions [46–49]. The third most common ST in this material was ST131, which was isolated from dogs only. This ST has been described as causing problematic infections in humans and has also been found in companion animals [3, 50]. Together, this indicates that numerous variants of ESBL/pAmpC producing *E. coli* can colonise and cause infections in cats and dogs, and that such bacteria can also spread between humans and cats and dogs.

On two occasions, cats in the same household were found to carry closely related isolates. The presence of similar strains from the same household could indicate that the strains were spreading within the household, or that the bacterial source was the same for the cats.

For the multiple isolates sampled from one individual, all but one were closely genetically related. These findings indicate that cats and dogs can harbour ESBL/AmpC-producing bacteria for a long time; in this study, the longest detected duration of carriage was nine and 15 months, respectively.

As a retrospective study of isolates that were sent for confirmation of ESBL/pAmpC-producing traits in bacteria, the shortage of clinical information for the included animals is a limitation. A prospective approach would allow for the collection of predetermined clinical data, as well as the outcomes of individuals, which would enable a deeper analysis of the results. The incidence of ESBL/pAmpC-producing bacteria in dogs and cats cannot be estimated based on the results of this study. All ESBL/pAmpC-producing bacteria do not cause clinical disease, and for evaluation of carrier status, a study design including sampling animals without clinical signs of disease is needed. By using data from a well-known laboratory where confirmation of ESBL/pAmpC-production is performed without extra cost, there is a good chance that most of the confirmed cases in Sweden during this period were included in the study. However, some isolates might never have been sent for confirmation and would therefore be missing.

## Conclusions

There were more isolates with ESBL/pAmpC-producing bacteria from dogs than from cats. The majority of the isolates were sampled from the urinary tract from both species and *E. coli* was the most common bacteria. A variety of sequence types and ESBL/pAmpC-genes were detected. Among the dogs, ST131 and ST372 were the dominant strains, and *bla*_CMY-2_ and *bla*_CTX-M-15_ were the dominant genes. Among the cats, ST73 was the dominant strain and *bla*_CTX-M-15_ the dominant gene.

Monitoring the resistance patterns and genetic relationships of bacteria over time is important for following the results of measures taken to reduce resistance. Combining knowledge of regional resistance patterns with treatment guidelines is valuable for veterinary clinicians to ensure appropriate antibiotic usage.

## Data Availability

The datasets generated and analysed during the current study are available from the corresponding author on reasonable request.
